# Supplementary material for: Regulatory T Cells Decreased during Recovery from Mild COVID-19
Source: Viruses. 2022 Jul 30;14(8):1688. doi: 10.3390/v14081688 (PMC9415862; doi:10.3390/v14081688)
Supplement: Supplementary file 1 [file viruses-14-01688-s001.zip › viruses-1793616-supplementary.pdf]

Supplementary

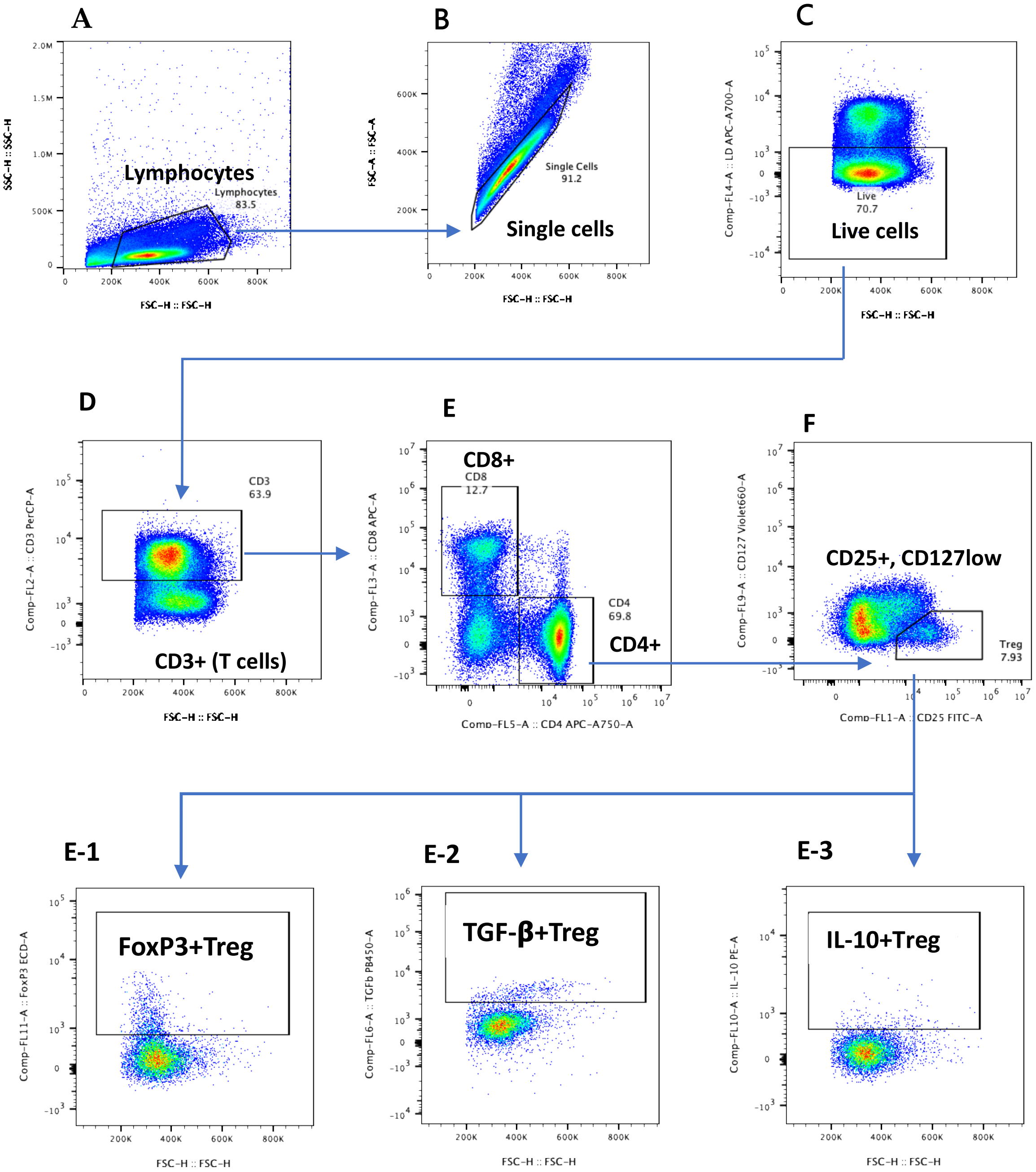

Figure S1. Gating strategy for finding Treg and cytokine producing Tregs. (A) All cells were identified as the lymphocytes by FSC-H and SSC-H. (B) Single lymphocytes were next selected from the panel. (C) Live cells presented next to the single cells panel. (D) Live cells were selected to identify T cells by CD3 positive population. (E) CD3 is a group of T cell which its subpopulation included of CD8 and CD4. (F) Tregs is a subpopulation of CD4+ identified by CD25+ and CD127 low as the surface markers. (E-1) were gated to identify Tregs' transcriptional factor FoxP3. (E-2) and (E-3) were demonstrated the cytokine producing Tregs, TGF-**β** and IL-10, respectively.

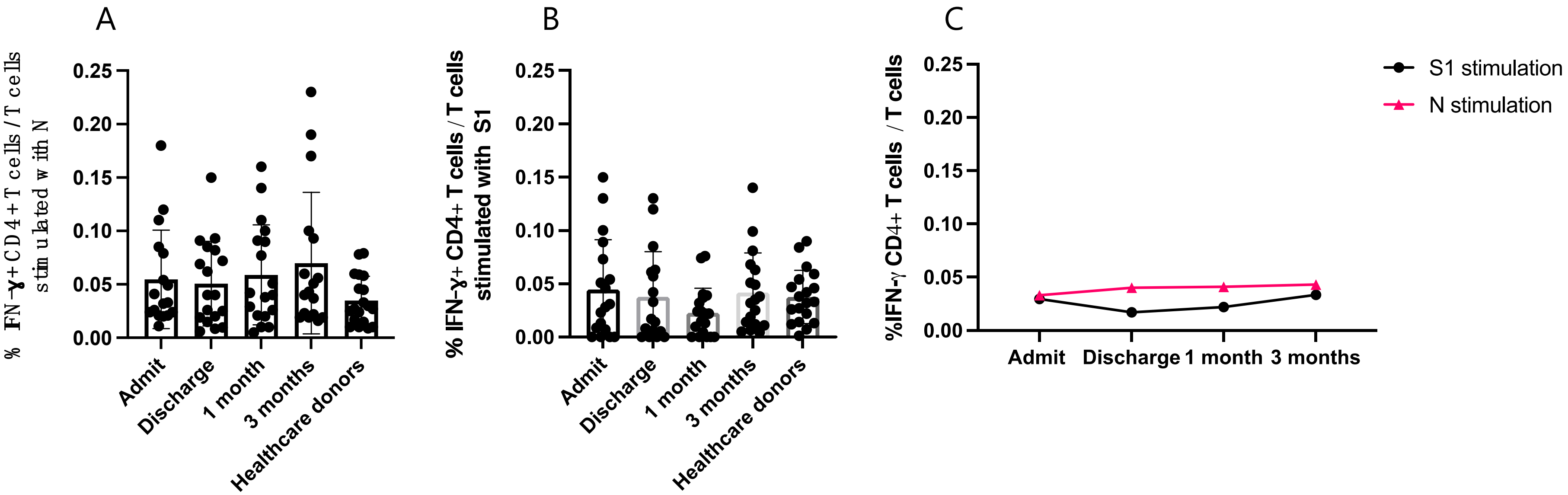

Figure S2. IFN-γ+ CD4+T-cell responses in COVID-19 patients. (A) and (B) are the percentage of IFN-γ +CD4+ T cells in T-cell population stimulated by SARS-CoV-2 N and S1 peptides. (C) Percentage IFN-γ+ CD4+ T cells in T-cell population in the form of kinetic change. The results are presented as median with 95% CI. Statistical significance of differences between groups was determined using Kruskal–Wallis, followed by Dunn's multiple comparison test.

Supplementary Table S1. Fluorochrome conjugated antibodies for flow cytometry analysis.

Antibody	Fluorochrome	Dilution	Clone	Cat no.	Source
FSC	-	-	-	-	-
SSC	-	-	-	-	-
CD3	PerCP	1:50	SP34-2	552851	BD Horizon
CD4	APC-H7	1:200	SK3	641398	BD
CD8	APC	1:200	SK1	340584	BD
CD25	BB515 (FITC)	1:100	2A3	554467	BD Horizon
CD127	BV650	1:100	HIL-7R-M21	563225	BD Horizon
L/D	FVS700	1:1000	-	564997	BD
INF-γ	PE-Cy7	1:100	B27	557643	BD pharmingen
TGF-β	BV-421	1:100	TW4-9E7	562962	BD Horizon
IL-10	PE	1:100	JES3-19F1	559330	BD pharmingen
FoxP3	PE-CFS594	1:100	236A/E7	563955	BD Horizon

Supplementary Table S2. Amino acid sequences of SARS-CoV-2 N peptides.

Peptide number	Amino acid start position	Sequence
1	1	MSDNGPQNQRNAPRI
2	5	GPQNQRNAPRITFGG
3	9	QRNAPRITFGGPSDS
4	13	PRITFGGPSDSTGSN
5	17	FGGPSDSTGSNQNGE
6	21	SDSTGSNQNGERSGA
7	25	GSNQNGERSGARSKQ
8	29	NGERSGARSKQRRPQ
9	33	SGARSKQRRPQGLPN
10	37	SKQRRPQGLPNNTAS
11	41	RPQGLPNNTASWFTA
12	45	LPNNTASWFTALTQH
13	49	TASWFTALTQHGKED
14	53	FTALTQHGKEDLKFP
15	57	TQHGKEDLKFPRGQG
16	61	KEDLKFPRGQGVPIN
17	65	KFPRGQGVPINTNSS
18	69	GQGVPINTNSSPDDQ
19	73	PINTNSSPDDQIGYY
20	77	NSSPDDQIGYYRRAT
21	81	DDQIGYYRRATRRIR
22	85	GYYRRATRRIRGGDG
23	89	RATRRIRGGDGKMKD
24	93	RIRGGDGKMKDLSPR
25	97	GDGKMKDLSPRWYFY
26	101	MKDLSPRWYFYYLGT
27	105	SPRWYFYYLGTGPEA

Peptide number	Amino acid start position	Sequence
28	109	YFYYLGTGPEAGLPY
29	113	LGTGPEAGLPYGANK
30	117	PEAGLPYGANKDGII
31	121	LPYGANKDGIIWVAT
32	125	ANKDGIIWVATEGAL
33	129	GIIWVATEGALNTPK
34	133	VATEGALNTPKDHIG
35	137	GALNTPKDHIGTRNP
36	141	TPKDHIGTRNPANNA
37	145	HIGTRNPANNAAIVL
38	149	RNPANNAAIVLQLPQ
39	153	NNAAIVLQLPQGTTL
40	157	IVLQLPQGTTLPKGF
41	161	LPQGTTLPKGFYAEG
42	165	TTLPKGFYAEGSRGG
43	169	KGFYAEGSRGGSQAS
44	173	AEGSRGGSQASSRSS
45	177	RGGSQASSRSSSRSR
46	181	QASSRSSSRSRNSSR
47	185	RSSSRSRNSSRNSTP
48	189	RSRNSSRNSTPGSSR
49	193	SSRNSTPGSSRGTSP
50	197	STPGSSRGTSPARMA
51	201	SSRGTSPARMAGNGG
52	205	TSPARMAGNGGDAAL
53	209	RMAGNGGDAALALLL
54	213	NGGDAALALLLLDRL
55	217	AALALLLLDRLNQLE
56	221	LLLLDRLNQLESKMS
57	225	DRLNQLESKMSGKGQ
58	229	QLESKMSGKGQQQQG
59	233	KMSGKGQQQQGQTVT
60	237	KGQQQQGQTVTKKSA
61	241	QQGQTVTKKSAAEAS
62	245	TVTKKSAAEASKKPR
63	249	KSAAEASKKPRQKRT
64	253	EASKKPRQKRTATKA
65	257	KPRQKRTATKAYNVT
66	261	KRTATKAYNVTQAFG
67	265	TKAYNVTQAFGRRGP
68	269	NVTQAFGRRGPEQTQ
69	273	AFGRRGPEQTQGNFG
70	277	RGPEQTQGNFGDQEL
71	281	QTQGNFGDQELIRQG
72	285	NFGDQELIRQGTDYK

Peptide number	Amino acid start position	Sequence
73	289	QELIRQGTDYKHWPQ
74	293	RQGTDYKHWPQIAQF
75	297	DYKHWPQIAQFAPSA
76	301	WPQIAQFAPSASAFF
77	305	AQFAPSASAFFGMSR
78	309	PSASAFFGMSRIGME
79	313	AFFGMSRIGMEVTPS
80	317	MSRIGMEVTPSGTWL
81	321	GMEVTPSGTWLTYTG
82	325	TPSGTWLTYTGAIKL
83	329	TWLTYTGAIKLDDKD
84	333	YTGAIKLDDKDPNFK
85	337	IKLDDKDPNFKDQVI
86	341	DKDPNFKDQVILLNK
87	345	NFKDQVILLNKHIDA
88	349	QVILLNKHIDAYKTF
89	353	LNKHIDAYKTFPPTE
90	357	IDAYKTFPPTEPKKD
91	361	KTFPPTEPKKDKKKK
92	365	PTEPKKDKKKKADET
93	369	KKDKKKKADETQALP
94	373	KKKADETQALPQRQK
95	377	DETQALPQRQKKQQT
96	381	ALPQRQKKQQTVTLL
97	385	RQKKQQTVTLLPAAD
98	389	QQTVTLLPAADLDDF
99	393	TLLPAADLDDFSKQL
100	397	AADLDDFSKQLQQSM
101	401	DDFSKQLQQSMSSAD
102	405	KQLQQSMSSADSTQA

Supplementary Table S3. Amino acid sequences of SARS-CoV-2 S1 peptides.

**Peptide number**	**Amino acid start position**	**Sequence**
1	1	MFVFLVLLPLVSSQC
2	6	VLLPLVSSQCVNLTT
3	11	VSSQCVNLTTRTQLP
4	16	VNLTTRTQLPPAYTN
5	21	RTQLPPAYTNSFTRG
6	26	PAYTNSFTRGVYYPD
7	31	SFTRGVYYPDKVFRS
8	36	VYYPDKVFRSSVLHS
9	41	KVFRSSVLHSTQDLF
10	46	SVLHSTQDLFLPFFS

Peptide number	Amino acid start position	Sequence
11	51	TQDLFLPFFSNVTWF
12	56	LPFFSNVTWFHAIHV
13	61	NVTWFHAIHVSGTNG
14	66	HAIHVSGTNGTKRFD
15	71	SGTNGTKRFDNPVLP
16	76	TKRFDNPVLPFNDGV
17	81	NPVLPFNDGVYFAST
18	86	FNDGVYFASTEKSNI
19	91	YFASTEKSNIIRGWI
20	96	EKSNIIRGWIFGTTL
21	101	IRGWIFGTTLDSKTQ
22	106	FGTTLDSKTQSLLIV
23	111	DSKTQSLLIVNNATN
24	116	SLLIVNNATNVVIKV
25	121	NNATNVVIKVCEFQF
26	126	VVIKVCEFQFCNDPF
27	131	CEFQFCNDPFLGVYY
28	136	CNDPFLGVYYHKNNK
29	141	LGVYYHKNNKSWMES
30	146	HKNNKSWMESEFRVY
31	151	SWMESEFRVYSSANN
32	156	EFRVYSSANNCTFEY
33	161	SSANNCTFEYVSQPF
34	166	CTFEYVSQPFLMDLE
35	171	VSQPFLMDLEGKQGN
36	176	LMDLEGKQGNFKNLR
37	181	GKQGNFKNLREFVFK
38	186	FKNLREFVFKNIDGY
39	191	EFVFKNIDGYFKIYS
40	196	NIDGYFKIYSKHTPI
41	201	FKIYSKHTPINLVRD
42	206	KHTPINLVRDLPQGF
43	211	NLVRDLPQGFSALEP
44	216	LPQGFSALEPLVDLP
45	221	SALEPLVDLPIGINI
46	226	LVDLPIGINITRFQT
47	231	IGINITRFQTLLALH
48	236	TRFQTLLALHRSYLT
49	241	LLALHRSYLTPGDSS
50	246	RSYLTPGDSSSGWTA
51	251	PGDSSSGWTAGAAAY
52	256	SGWTAGAAAYYVGYL
53	261	GAAAYYVGYLQPRTF
54	266	YVGYLQPRTFLLKYN
55	271	QPRTFLLKYNENGTI

Peptide number	Amino acid start position	Sequence
56	276	LLKYNENGTITDAVD
57	281	ENGTITDAVDCALDP
58	286	TDAVDCALDPLSETK
59	291	CALDPLSETKCTLKS
60	296	LSETKCTLKSFTVEK
61	301	CTLKSFTVEKGIYQT
62	306	FTVEKGIYQTSNFRV
63	311	GIYQTSNFRVQPTES
64	316	SNFRVQPTESIVRFP
65	321	QPTESIVRFPNITNL
66	326	IVRFPNITNLCPFGE
67	331	NITNLCPFGEVFNAT
68	336	CPFGEVFNATRFASV
69	341	VFNATRFASVYAWNR
70	346	RFASVYAWNRKRISN
71	351	YAWNRKRISNCVADY
72	356	KRISNCVADYSVLYN
73	361	CVADYSVLYNSASFS
74	366	SVLYNSASFSTFKCY
75	371	SASFSTFKCYGVSPT
76	376	TFKCYGVSPTKLNDL
77	381	GVSPTKLNDLCFTNV
78	386	KLNDLCFTNVYADSF
79	391	CFTNVYADSFVIRGD
80	396	YADSFVIRGDEVRQI
81	401	VIRGDEVRQIAPGQT
82	406	EVRQIAPGQTGKIAD
83	411	APGQTGKIADYNYKL
84	416	GKIADYNYKLPDDFT
85	421	YNYKLPDDFTGCVIA
86	426	PDDFTGCVIAWNSNN
87	431	GCVIAWNSNNLDSKV
88	436	WNSNNLDSKVGGNYN
89	441	LDSKVGGNYNYLYRL
90	446	GGNYNYLYRLFRKSN
91	451	YLYRLFRKSNLKPFE
92	456	FRKSNLKPFERDIST
93	461	LKPFERDISTEIYQA
94	466	RDISTEIYQAGSTPC
95	471	EIYQAGSTPCNGVEG
96	476	GSTPCNGVEGFNCYF
97	481	NGVEGFNCYFPLQSY
98	486	FNCYFPLQSYGFQPT
99	491	PLQSYGFQPTNGVGY
100	496	GFQPTNGVGYQPYRV

Peptide number	Amino acid start position	Sequence
101	501	NGVGYQPYRVVVLSF
102	506	QPYRVVVLSFELLHA
103	511	VVLSFELLHAPATVC
104	516	ELLHAPATVCGPKKS
105	521	PATVCGPKKSTNLVK
106	526	GPKKSTNLVKNKCVN
107	531	TNLVKNKCVNFNFNG
108	536	NKCVNFNFNGLTGTG
109	541	FNFNGLTGTGVLTES
110	546	LTGTGVLTESNKKFL
111	551	VLTESNKKFLPFQQF
112	556	NKKFLPFQQFGRDIA
113	561	PFQQFGRDIADTTDA
114	566	GRDIADTTDAVRDPQ
115	571	DTTDAVRDPQTLEIL
116	576	VRDPQTLEILDITPC
117	581	TLEILDITPCSFGGV
118	586	DITPCSFGGVSVITP
119	591	SFGGVSVITPGTNTS
120	596	SVITPGTNTSNQVAV
121	601	GTNTSNQVAVLYQDV
122	606	NQVAVLYQDVNCTEV
123	611	LYQDVNCTEVPVAIH
124	616	NCTEVPVAIHADQLT
125	621	PVAIHADQLTPTWRV
126	626	ADQLTPTWRVYSTGS
127	631	PTWRVYSTGSNVFQT
128	636	YSTGSNVFQTRAGCL
129	641	NVFQTRAGCLIGAEH
130	646	RAGCLIGAEHVNNSY
131	651	IGAEHVNNSYECDIP
132	656	VNNSYECDIPIGAGI
133	661	ECDIPIGAGICASYQ
134	666	IGAGICASYQTQTNS
